# Splenic epithelial cyst mistaken with Hydatid cyst: A case report

**DOI:** 10.1016/j.ijscr.2018.10.011

**Published:** 2018-10-12

**Authors:** Youssef A. Sleiman, Ali Bohlok, Melody El-Khoury, Pieter Demetter, Marc Zalcman, Issam El Nakadi

**Affiliations:** a Service de Chirurgie, Institut Jules Bordet, Université Libre de Bruxelles (ULB), Belgium; b Service de Chirurgie, Université Libanaise, Beirut, Lebanon; c Service d’Anatomie Pathologique, Erasme, Université Libre de Bruxelles (ULB), Belgium; d Service de radiologie, Erasme, Université Libre de Bruxelles (ULB), Belgium

**Keywords:** Splenic epithelial cysts, Hydatid cyst, Spleen conserving surgery

## Abstract

Introduction: Cystic lesions of the spleen are infrequent and usually diagnosed incidentally. These lesions are rare and their differential diagnosis is very wide. Splenic epithelial cysts are the most common type of primary splenic cyst (4%). Surgical treatment is indicated for cysts larger than 5 cm or symptomatic. Nowadays, spleen preserving surgery is the gold standard treatment considering the immunologic role of the spleen and the increased risk of post-splenectomy infections.

Case presentation: A 17 year old girl presented to the outpatient clinic with moderate left upper quadrant abdominal pain of 2 weeks duration with loss of appetite and denied fever, chills, sweating and jaundice. Abdominal examination showed tender splenomegaly. The abdominal imaging (Ultrasound, CT SCAN, MRI) was suggestive of hydatid cyst of the spleen, for which she received Albendazole for 1 month and then operated by laparotomy with partial resection and un-roofing of the splenic cyst. The pathology report showed a splenic epithelial cyst (SEC).

Conclusion: SEC is a rare pathology that could mimic splenic hydatid cyst. The clinical and radiological pictures may be commonly misleading and non-conclusive. Definitive diagnosis is made on histopathology. Spleen conserving surgery, when possible, is the preferred modality for treatment.

## Highlights

• Cystic lesions of the spleen are rare pathology with epithelial cyst being the most common type.
• The radiologic imaging may be commonly misleading and non-conclusive and the definitive diagnosis is made on histopathology.
• Splenic epithelial cyst should be kept in the differential diagnosis of a splenic cyst along with hydatid disease.
• Surgical treatment is indicated for symptomatic cysts or those larger than 5 cm.

## 1 Introduction

Splenic cystic lesions are uncommon entity with an incidence rate of 0.07% reported in a review of 42,327 autopsies [1]. They are classified into true and false cysts based on the presence of cellular epithelial lining [2].True or primary cysts are epithelium-lined cysts and represent 25% of all splenic cysts. According to their etiology, they are classified as congenital (SEC), neoplastic or parasitic cysts [3]. SEC is benign, sporadic and occurs predominantly in females between the second and third decade of life [2,4]. The clinical presentation is nonspecific and it varies from asymptomatic occurrence to symptoms related to the size, location and the presence of cyst complications [5].

Diagnostic imaging modalities such as abdominal ultrasound, scan and MRI demonstrate easily the splenic cyst but are unable to differentiate SEC from other types of primary splenic cyst such as parasitic and neoplastic [5,6]. The definitive diagnosis is based on the histopathologic examination of the operative specimen [6].

We herein report an interesting case of large epithelial cyst with elevated serum total IgE level misdiagnosed as Hydatid cyst. This work has been reported in line with the SCARE criteria [7].

## 2 Case presentation

**Fig. 1:**
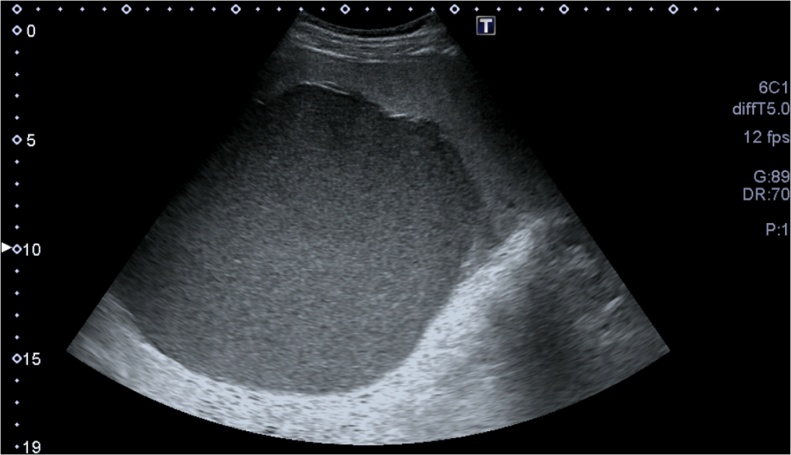
Abdominal ultrasound showing a large splenic cyst containing homogenous internal echoes/debris.

**Fig. 2:**
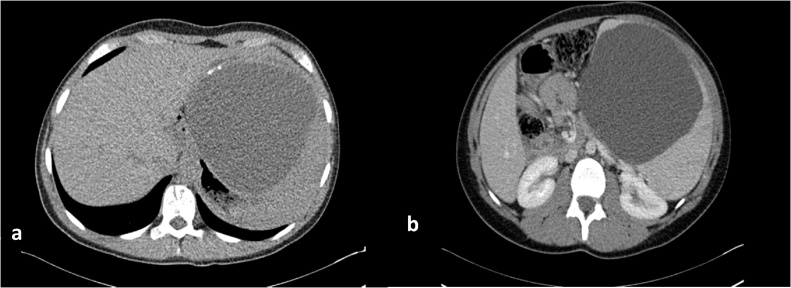
(a) Axial image of non-contrast CT abdomen shows the large splenic cyst with focal peripheral calcification in wall (White arrow), (b) contrast enhanced CT shows no peripheral enhancement, no floating membranes or enhancing solid component.

**Fig. 3:**
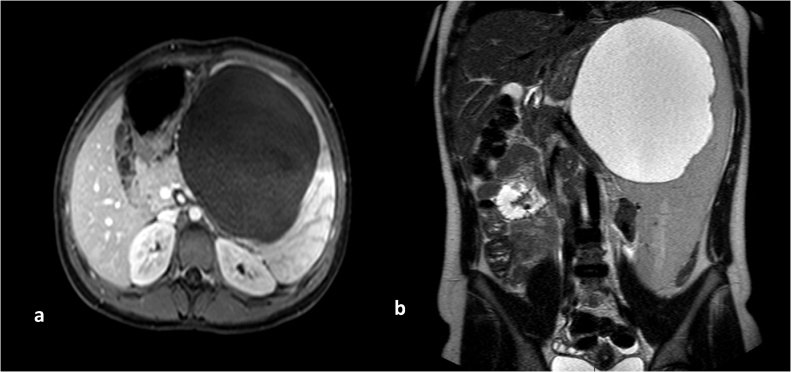
Axial T1 post contrast MR image shows a non-enhancing cystic lesion (3a), Coronal T2 image shows a hyper-intense fluid signal intensity splenic lesion (3b).

A 17 year old horse-rider girl was referred to our clinic for 2 weeks history of moderate continuous, crampy abdominal pain, starting in the epigastric region and shifted to the left upper quadrant. This pain was associated with fatigue, loss of appetite. Patient denies any nausea, vomiting, diarrhea, fever, night sweats. She reported a remoteleft shoulder pain with negative MRI. Her physical exam was positive for splenomegaly 9 cm below costal margins, and left upper quadrant tenderness, with no rebound tenderness. Her laboratory examination showed a Hg:12.8 g/dL, platelet: 124,000/mm^3^, WBC: 6500/mm^3^ with 62% neutrophils, 24% lymphocytes, and 7% eosinophils, platelet: 124,000/mm^3^, a CRP:0.78 mg/L. The Liver enzymes, Bilirubin, Albumin, LDH, the Chemistry panel were all in normal range. Abdominal ultrasound showed a large splenic cyst of 15 cm containing homogenous internal debris (Fig. 1). An abdominal computed tomography scan showed the same 15 cm splenic cyst with parietal calcifications, compressing the stomach, most likely of hydatid origin (Fig. 2a, b). Abdominal MRI showed unilocular splenic cyst hypo-intense T1, hyper-intense T2 (Fig. 3a, b). Differential diagnosis for described findings include; Splenic abscess, Hydatid cyst, epithelial cyst and post traumatic hemorrhage in pre-existing epithelial cyst. Based on clinical picture and endemic status for hydatid cyst differential can be narrowed.

Serologic test for Hepatitis B virus (HBV), hepatitis C virus (HCV), Cytomegalovirus (CMV), HIV, Toxoplasmosis, Entamoeba histolytica, Leishmania brazilensis, donovani, and EBV IgM were all negative. EBV IgG was elevated showing prior immunization. The immune-diffusion test for Echinococus multilocularis was negative. The Indirect hemagglutination test and the Elisa test for Echinococcus granulosus were also negative.

Nevertheless, due to an elevated IgE level: 317 kU/L, the patient was considered as having splenic hydatid cyst and was treated by albendazole PO with meals in a dose of 400 mg twice daily for 28 days, and received the anti-pneumococcal vaccine.

Due to the severe continuous pain, the large size, the risk of spontaneous rupture and the patient’s wishes to resume her hobby as a horse-rider as soon as possible, she was consented for operative exploration via a laparotomy incision for splenic cyst un-roofing.

Exploration was done, abdominal cavity was protected by hypertonic saline (3%NaCl) filled pads, cyst was punctured, 2 liters of dark green fluid was aspirated. Hypertonic saline was injected in the cyst, and then aspirated after 15 min. Un-roofing and partial resection was done afterward.

The postoperative course was un-eventful and the patient was discharged home on the post-operative day 5.

The pathology report showed stratified epithelium with fibro-inflammatory reaction in the pericystic zone compatible with splenic epithelial cyst.

The patient still symptom free after 5 years of follow up and her labs showed a WBC: 7700/mm^3^ normalization of eosinophils (2.5%).

## 3 Discussion

SEC are congenital true splenic cyst characterized by an epithelial lining. They are divided in 3 subgroups depend on the type of epithelial lining, Epidermoid cysts are covered with stratified squamous epithelial lining, Mesothelial cyst with cuboidal epithelial lining and Dermoid cyst with squamous epithelium with hair follicles, sebaceous glands and skin appendages [8]. The epidermoid subtype represents 10% of all SEC and they are strongly linked to elevated CA 19-9 level because inner epithelial cells secrete CA 19-9 [9]. By immunohistochemistry, the epidermoid cysts are CA 19-9, CEA and cytokeratin positive but show no immunoreactivity for calretinin; whereas mesothelial cysts are calretinin and cytokeratin positive but show no staining for CA 19-9 and CEA [10].

The clinical presentation of SEC is non-specific and it can be various depend on the size, location and the presence of complications. Uncomplicated cyst less than 8 cm in diameter are usually asymptomatic [11]. The increase in its size leads to distention of its capsule and therefore the development of pain and mainly in the left upper quadrant which is the most common symptom. Progressive symptoms are related to compression of adjacent organs including distension, early satiety, vomiting, flatulence, persistent cough, pleuritic pain and hydronephrosis due to local pressure on stomach, left hemi-diaphragm and left kidney respectively [12]. In very rare cases, complications of the cyst such as hemorrhage, infection or rupture induce peritoneal sign due to hemoperitoneum, peritonitis or even sepsis [9].

Regarding the pre-operative diagnosis of our case, the negative Echinococcosis detection test doesn’t rule out the hydatid disease and is present only in case of microscopic rupture [13]. Accordingly, the association of elevated IgE level made the diagnosis more difficult. Although elevated IgE is not specific for hydatid disease, but is present in a large subset of affected individuals [14].

Nowadays, the expansion of medical screening systems increases the incidental detection of splenic lesions especially for SEC [15]. Epithelial cysts appear as well defined, thin wall, liquid containing lesions on Ultrasound, CT scan and MRI. They can contain debris in case of intra-cystic bleed or infection [16]. Abdominal MRI has a higher sensitivity in the identification of the septa and calcification [12]. But all of these diagnostic modalities are unable to differentiate the SEC from other splenic cysts. The definitive diagnosis is established by the anatomo-pathologic examination [2].

Concerning the indication of surgical treatment, there is a limited data to determine the appropriate time to interfere. Traditionally, it has been recommended to treat symptomatic cysts, or when they are larger than 5 cm due to an increased risk of spontaneous rupture, hemoperitoneum, chemical peritonitis or abscess formation [17]. However, some studies described that small cysts may be also at risk of rupture after a simple trauma or heavy cough in infants [18].

There are multiple surgical treatment modalities including aspiration, marsupialization, cystectomy, cyst de-roofing, cyst de-capsulation, partial splenectomy and splenectomy [19,20].

Historically, open total splenectomy was the ideal surgical approach in front of SEC in order to decrease the risk of bleeding and complications from the cyst. Currently, with the awareness of immunologic role of the spleen and particularly in young age and the increased risk of post splenectomy sepsis, splenic preserving surgery with laparoscopic approach is advocated [19,20]. Laparoscopic de-roofing is reported to be effective treatment of splenic epithelial cyst but it carries a risk of recurrence of about 22% of which only 3% needs re-intervention [21]. The choice of surgical procedure depends on several factors like: the amount of remaining healthy splenic tissue, the size, number and location of the cyst in relation to the hilum, pathogenesis of the cyst and patient’s age [2].

In conclusion, epithelial splenic cyst is a rare entity, usually diagnosed incidentally in asymptomatic patients. Symptoms when present are related to size and location. SEC can be misdiagnosed as hydatid cyst. Imaging can be helpful in the diagnosis but cannot differentiate it from the other types of primary cyst. The definitive diagnosis is based on pathologic examination of operative specimen. SEC is best treated by parenchymal sparing surgery.

## Conflicts of interest

We have no conflict of interest to declare.

## Funding

No funding source.

## Ethical approval

The submitted article is a case report, ethical approval has been exempted by our institution.

## Consent

A Written informed consent was obtained from the patient for surgery and potential publication of this case report and any accompanying images.

## Authors contribution

Youssef Sleiman and Ali Bohlok wrote the manuscript. Melody El-Khoury assisted in large part of the literature review and revised the manuscript for correction before submission. Marc Zalcman did the imaging diagnosis, and wrote the imaging part of the manuscript. Issam El Nakadi and operated the patient, wrote the part about the detailed operative technique and revised the manuscript for correction before submission. Peter Demetter and did the pathologic examination of the operative specimen and explained the pathologic diagnosis in the manuscript. All authors have read and approved the manuscript before submission to your journal.

## Registration of research studies

Not applicable.

## Guarantor

Dr Issam El Nakadi.

## Availability of data and materials

The data sets supporting the conclusions of this article are included within the article.

## Compliance with ethics guidelines

All procedures reported here were in accordance with the ethical standards of the Institut Jules-Bordet Committee and with the 1964 Declaration of Helsinki and its later amendments or comparable ethical standards. Informed consent was obtained from all the subject of the case report included in the study.

## Provenance and peer review

Not commissioned, externally peer reviewed.
